# Improving LC-MS analysis of human milk B-vitamins by lactose removal

**DOI:** 10.1016/j.jchromb.2021.122968

**Published:** 2021-10-15

**Authors:** Daniela Hampel, Setareh Shahab-Ferdows, John W. Newman, Lindsay H. Allen

**Affiliations:** a USDA, ARS Western Human Nutrition Research Center, 430 West Health Sciences Drive, Davis, CA 95616, USA; b Department of Nutrition, University of California, One Shields Ave, Davis, CA 95616, USA

**Keywords:** ACN, acetonitrile, B, biotin, CAF, caffeine, EtOAc, ethyl-acetate, EtOH, ethanol, FAD, flavin adenine dinucleotide, IPA, 2-propanol, IS, internal standard, LSE, liquid-solid extraction, MeOH, methanol, MP, mobile phase, NAM, nicotinamide, PA, pantothenic acid, PL, pyridoxal, PN, pyridoxine, PPT, protein precipitation, R, riboflavin, RP, reversed-phase, T, thiamin, B-vitamins, Lactose, Human milk, UPLC-MS/MS

## Abstract

Our previously reported, first validated, UPLC-MS/MS-based simultaneous analysis of five human milk B-vitamins revealed severe matrix effects. High levels of endogenous lactose fouled the electrospray ionization source affecting the analysis. We evaluated solid-phase extraction (SPE), liquid–solid extraction (LSE), protein precipitation (PPT), and liquid chromatography effluent diversion for lactose-removal. SPE failed to separate lactose from vitamins; LSE using 2-propanol reduced lactose and vitamin recoveries. PPT-solvent, milk volume, and reconstitution solvent influenced flavin adenine dinucleotide, pyridoxal and nicotinamide recoveries. Using an optimized LC-gradient enabled chromatographic separation of lactose from vitamins and its removal using a post-column switch-valve. Only 40 µL milk was subjected to methanol-PPT and non-polar matrix removal by methyl *tert*-butyl ether. B-vitamin recoveries were established (81.9–118.6%; CV ≤ 11.9%; precision: 4.9–13.7%) with greatly reduced matrix effects, and improved process efficiency, and recovery.

## Highlights

• Lactose in human milk affects B-vitamin analysis by fouling the ion source.
• Typical extraction methods are inefficient for lactose removal.
• Chromatographic separation from analytes enables lactose mechanical removal.

## 1 Introduction

Human milk B-vitamins have been a topic of investigation for decades [1], [2]. Analytical methods were usually adapted from those developed for blood, plasma, or urine samples [1], matrices with negligible amounts of sugar and fat, unlike human milk [3], [4] (Supplemental Fig. 1); and proper validation for the intricate milk matrix has rarely been described.

B-vitamins differ in their chemical and physical properties. Associations with other milk constituents complicate their simultaneous analysis; some procedures benefit certain B-vitamins while others degrade under the same conditions [1], [2]. Their light, temperature, and pH sensitivities demand rapid but gentle handling. We reported the first validated method for simultaneously analyzing several B-vitamins in human milk by ultra-performance liquid-chromatography-*tandem* mass-spectrometry (UPLC-MS/MS) [2], revealing severe matrix effects, and recently described methods [5], [6] continue to lack focus on matrix effects, recovery, and process efficiency, and how to overcome these limitations.

LC-MS offers superior sensitivity, selectivity, and specificity, but electrospray ionization suppression or enhancement can affect precision and accuracy [7], [8], [9], [10], with the most challenging interferences caused by co-eluting matrix components [9]. Human milk contains about 7% lactose, a polar disaccharide [3], and reported sample preparations [2], [5], [6] are ineffective in its removal. If not excluded, lactose can rapidly foul the ionization source, decreasing method robustness and impacting detection limits over the course of the analysis. We evaluated solid-phase extraction (SPE), liquid–solid extraction (LSE), and protein precipitation (PPT) for lactose removal, a crucial but missing purification step in previously reported methods, to compare matrix effects, process efficiency, and analyte recovery with previously published reports.

## 2 Materials and methods

### 2.1 Chemicals and materials

Details of chemicals and materials used in this study are listed in Supplemental Table 1.

### 2.2 UPLC-MS/MS system

This system consisted of an ACQUITY UPLC (Waters; Milford, MA, USA) coupled to an API 4000QTRAP-MS (Sciex; Foster City, CA, USA), connected by a two-position switching valve (Valco Instruments, Houston, TX, USA) between UPLC-outlet and MS-inlet. 3–10 µL sample was injected onto an ACQUITY UPLC® HSS T3 column (2.1 × 100 mm, 1.8 µm) protected by an ACQUITY UPLC® HSS T3 VanGuard^TM^ pre-column (2.1 × 5 mm, 1.8 µm; Waters) in reversed-phase (RP) mode (Supplemental Table 3). Starting at 1.8 min, the switch valve diverted the effluent to the detector, enabling a lactose-free detection of the B-vitamins (Supplemental Figures 2 and 3). MS-acquisition was optimized for positive ion mode electrospray ionization (ESI) multiple reaction monitoring (MRM) with Q1/Q3 unit (Supplemental Tables 2 and 3).

### 2.3 HPLC-MS/Ms

An Alliance 2695 HPLC coupled to a Micromass Micro Quattro MS (Waters) was used to evaluate SPE, LSE, and PPT, and to optimize chromatography to resolve lactose and B-vitamins. Analytes were injected onto an Atlantis T3 column (3 µm, 2.1x75mm; Waters), protected by a BDS-hypersil C18 Javelin-guard (3 µm, 3 x 20mm; Thermo Fisher Scientific, Waltham, MA, USA) in RP-mode (Supplemental Table 3). Vitamins were detected in ESI in positive mode in MRM with Q1/Q3 unit resolution (Supplemental Tables 2 and 3). Lactose was detected in negative ESI in full-scan mode (*m*/*z* = 50–400; Supplemental Table 3).

### 2.4 Human milk samples

Human milk (HMP) was pooled from healthy donors from Vancouver, BC, Canada, and used as for the all experiments as described. Aliquots were stored at −70 °C in amber tubes.

### 2.5 Preparation of standards

All external and internal aqueous standard solutions were prepared under dim light on ice and in amber glass vials (Supplemental Table 4). Stable isotope labeled vitamins were used as internal standards for quantitation, ^13^C_3_-caffeine was used as a system control to adjust for potential system fluctuation [2]. The stable-isotope internal standards were combined to an aqueous master mix (1 µg/mL; IS) of which 10 µL was added to each sample, test-mix, or lactose solution.

### 2.6 Solid phase extraction

50 µL aqueous standard test-mix (thiamin, riboflavin, FAD, nicotinamide, and pyridoxal: 500 ng/mL) and 150 µL methanol (MeOH) were combined (mock sample) to mimic PPT of milk as previously described [2]. A 5% aqueous lactose solution was treated identically and applied to a separate SPE cartridge. The OASIS® HLB sorbent was chosen due to its all-purpose, strongly hydrophilic, water-wettable polymer with a hydrophilic-lipophilic balance. After conditioning, the mock sample or lactose solution were applied followed by a wash step prior to a stepwise elution (Table 1). The collected fractions were adjusted to 500 µL for analysis. This served as a preliminary experiment with no replicates.

**Table 1:** SPE elution profiles for B-vitamins and lactose and analyte recovery after liquid–solid extraction (LSE) of B-vitamins from neat standard mix and human milk.

**SPE^1^**	**Lactose**	**Thiamin^2^ [%]**	**Riboflavin**	**FAD**	**NAM**	**Pyridoxal**	**Caffeine**
***Condition NH_4_F/ACN***							
Flow through	70	45	31	37	43	38	21
Wash	30	54	28	51	56	58	74
95/5	n.d.	0.5	7.5	5.5	1.4	3.3	3.6
90/10	n.d.	0.1	15	6.4	n.d.	1.0	1.8
85/15	n.d.	0.1	8.2	n.d.	n.d.	n.d.	n.d.
80/20	n.d.	> 0.1	5.5	n.d.	n.d.	n.d.	n.d.
50/50	n.d.	> 0.1	5.4	n.d.	n.d.	n.d.	n.d.
70/30 _ACN/MeOH_	n.d.	n.d.	n.d.	n.d.	n.d.	n.d.	n.d.
***Condition AA/ACN***							
Flow through	81	96	1.1	n.d.	62	75	n.d.
Wash	19	3.4	1.9	n.d.	35	25	n.d.
95/5	n.d.	n.d.	4.3	n.d.	2.7	0.3	8.9
90/10	n.d.	n.d.	45	8.9	n.d.	> 0.1	82
85/15	n.d.	n.d.	36	79	n.d.	n.d.	9.1
80/20	n.d.	n.d.	4.9	11	n.d.	n.d.	n.d.
50/50	n.d.	n.d.	23	1.2	n.d.	n.d.	n.d.
100_MeOH_	n.d.	1.1	2.8	n.d.	n.d.	n.d.	n.d.
100_ACN_	n.d.	n.d.	1.7	n.d.	n.d.	n.d.	n.d.
**LSE^2^**							
Acetone	----	52	44	n.d.	71	40	82
EtOAc	----	0.1	70	1.4	78	9.0	85
IPA	8.3	80	87	57	81	90	85
MeOH	95	82	87	70	79	89	73
***Human milk^3^***							
Average	----	14	17	17	33	64	99
SD	----	± 2.2	± 0.3	± 2.8	± 1.1	± 6.3	± 13

^1^Stepwise elution ratios of indicated solvent system (NH_4_F/ACN or AA/ACN; v,v). The cartridges were conditioned with ACN and either 10 mM NH_4_-formate_aq_ and ACN (95/5, v/v) or 0.1% acetic acid_aq_ and ACN (95/5, v/v). Elution profile expressed as percent [%] of the combined peak areas for all fractions collected in each SPE experiment. ^2^Analyte recovery expressed as percent [%] of the neat standard. ^4^Lactose was only tested using IPA and MeOH. ^3^Milk samples (in duplicates) were only subjected to IPA due to the superior lactose removal. The analyte recovery is expressed as percent [%] based on milk samples prepared without LSE.

AA: 0.1% acetic acid, ACN: acetonitrile, EtOAc: ethyl-acetate, FAD: flavin adenine dinucleotide, IPA: 2-propanol, MeOH: methanol, FAD: flavin adenine dinucleotide, NAM: nicotinamide, NH_4_F: 10 mM NH_4_-formate, MeOH: methanol.

### 2.7 Liquid-Solid extraction

LSE was evaluated as an additional clean-up step. Acetone, ACN, ethanol (EtOH), ethyl-acetate (EtOAc), 2-propanol (IPA), and MeOH were tested. 40 µL of the standard test-mix were evaporated to dryness and subjected to LSE using 100 µL solvent. The supernatant obtained was evaporated and reconstituted with 100 µL initial mobile phase. The sample was back-extracted with MTBE to mimic the removal of non-polar matrix. LSE-conditions were tested with 40 µL lactose solution when all tested vitamins were recovered > 50%. LSE using HMP was done with solvents efficiently removing lactose while maintaining analyte recoveries > 50%. Results obtained were compared to those from HMP prepared without LSE (Table 1).

### 2.8 Protein precipitation

Different PPT-solvents were tested using a modified Taguchi method [11]. MeOH, acetone, and IPA were tested as PPT-solvents. ACN caused low FAD and nicotinamide recovery in human milk [2] and was excluded. Additional parameters tested included sample volume and reconstitution solvent (Table 2). The sample preparation followed the same principle as described above.

**Table 2:** Modified Taguchi method for optimizing PPT procedures.

**Sample**	**PPT**		**Milk**	**Reconstitution**		**T**	**R**	**FAD**	**NAM**	**PL**
	Solvent	Volume [µL]	Volume [µL]	Solvent^1^	Ratio [v,v]	Adjusted peak area response by milk volume and IS [%]				
1	MeOH	75	25	A/B	95/5	0.56	6.1	0.50	1.9	7.1
2	MeOH	150	50	A/B	90/10	0.53	5.6	0.56	0.96	8.9
3	MeOH	300	100	A/C	90/10	0.54	5.6	0.55	0.80	9.8
4	Acetone	75	25	A/C	90/10	0.58	6.2	0.15	2.96	7.1
5	Acetone	150	50	A/B	95/5	0.57	6.3	0.18	1.89	9.3
6	Acetone	300	100	A/B	90/10	0.50	5.6	0.10	0.97	13.3
7	IPA	75	25	A/B	90/10	0.58	6.2	0.19	2.8	7.2
8	IPA	150	50	A/C	90/10	0.56	6.3	0.17	1.73	7.8
9	IPA	300	100	A/B	95/5	0.52	5.6	0.13	1.06	11.3
AVG						0.55	5.9	0.28	1.68	9.1
SD						0.03	0.32	0.19	0.81	2.13
CV						5.1	5.3	68.1	48.1	23.5

1 A: 10 mM NH_4_-formate_aq_; B: ACN; C: MeOH. ACN: acetonitrile, IPA: 2-propanol, MeOH: methanol.

### 2.9 Sample preparation

Samples were prepared based on our previous report [2] and optimized based on results obtained here. 10 µL IS and 40 µL sample were combined, and 150 µL MeOH was added for PPT, followed by vortexing for 2 min. After centrifugation (10 min, 4 °C, 19098 × g SORVALL® LEGEND RT, Asheville, NC), the supernatant was transferred into a 4 mL amber glass vial to dry under a gentle nitrogen stream. The residue was reconstituted in 100 µL 10 mM NH_4_-formate_aq_ /ACN 99/1 (v/v) containing ^13^C_3_-caffeine-trimethyl (100 ng/mL) and transferred into a fresh 1.5 mL amber centrifuge tube on ice, containing 200 µL MTBE. After mixing for 30sec, the two phases were separated by centrifugation (3 min, 4 °C, 19098 × g). The lower phase (sample extract) was filtered (0.1 µm) and analyzed.

### 2.10 Quality control

An aqueous eight-point standard curve (1–500 µg/L; nicotinamide: 5–2500 µg/L; Ca-pantothenate: 15–7500 µg/L) and a reagent blank were included with each batch of the experiments. The analytes were quantified using the respective trendlines (f(x) = m*x + b; m: slope, b: intercept), as determined by ratio response of analyte to IS.

### 2.11 Analyte recovery

A 3-level standard addition experiment on 5 different days determined recovery rates using HMP with unknown B-vitamin concentrations. 5 replicates for each level (0, 5, 10, and 15 µL of a stock solution [500 µg/L; nicotinamide: 2.5 mg/L, Ca-pantothenate: 7.5 mg/L]) were added to HMP. Recovery rates for each vitamer were calculated as follows:

R [%] = [Δ(C_analyzed_ – C_endogenous_)*100]/C_StdAdd_

### 2.12 Matrix effects

Matrix effects were assessed in HMP using the stable isotope-labeled IS (100 ng/mL). Five sets were prepared for calculating matrix effects (ME), recovery (RE) of the extraction procedure, and overall process efficiency (PE), according to Matuszewski et al. [2], [10]:

ME [%] = Set2*100/Set1

RE [%] = Set3*100/Set1

PE [%] = Set3*100/Set2 = (ME*RE)/100

Set 1 represented the neat, aqueous standard solution, sets 2 and 3 were prepared as described but adding the standards either pre- (Set 3) or post- sample preparation (Set 2). Three replicates were injected for each set and injection volumes (3, 5, and 10 µL). The latter was used to assess effects caused by amount of injected matrix.

### 2.13 Analyte stability during sample preparation

Five sequential sets of 24 samples were prepared throughout each day, each set including 2 HMPs. On the first day, an HMP aliquot of 600 µL was thawed with the first sample set, stored on ice, and used throughout the day as a source for the 2 HMPs per set. On day 2, two 40 µL aliquots of frozen HMP were thawed with each sample set prior to sample preparation. Both HMP aliquots were analyzed at the beginning and at the end of the set. The B-vitamin concentrations were compared within and between the sets.

### 2.14 Statistical analysis

Descriptive statistics (mean, standard deviation, coefficient of variation) were carried out using Microsoft Excel 2016 (Microsoft Corporation, Redmond WA, USA). Difference in ME, PE, and RE by injection volume were examined by generalized linear models. Student’s *t*-test was used to evaluate matrix effects to already published data, using the results obtained with the same injection volume (10 µL). P-values below 0.05 were considered significant.

## 3 Results and discussion

### 3.1 Solid-phase extraction

SPE using NH_4_-formate/ACN resulted in minimal column-retention of all tested analytes (Table 1). Flow-through and column wash contained most amounts for nearly all vitamins, and lactose. Only riboflavin was detected in several fractions (5–15%). Using acetic acid, thiamin, nicotinamide, pyridoxal, and lactose continued to co-elute; riboflavin, FAD, and caffeine eluted mostly at 90/10–75/15 (acetic acid/ACN, v/v). However, none of the tested conditions separated lactose from all analytes.

### 3.2 Liquid-solid extraction

ACN and EtOH were unsuitable for FAD and therefore excluded. Using EtOAc resulted in low recoveries for thiamin, FAD, and pyridoxal, and FAD was not detected using acetone (Table 1). Nicotinamide and ^13^C-caffeine were recovered > 70% in all solvents. Only MeOH and IPA produced good overall recoveries and were applied to the lactose solution, which revealed a > 90% recovery with MeOH but < 10% when using IPA. Thus, HMP was only tested using IPA. When compared to HMP without LSE, thiamin, riboflavin, and FAD showed < 20%, nicotinamide ∼ 30%, and pyridoxal ∼ 60%, recovery. ^13^C-caffeine was not affected (99% recovery). Thus, LSE was ineffective in lactose removal while maintaining acceptable analyte recoveries.

### 3.3 Protein precipitation

Thiamin and riboflavin were not affected by choice of PPT-solvent, sample volume, or reconstitution solvent (Table 2). Nicotinamide was negatively affected by sample matrix, while the opposite was true for pyridoxal. Acetone and IPA were superior for nicotinamide and pyridoxal, while FAD revealed a higher response with MeOH, indicating that optimal conditions are analyte-depended. Analyte recoveries after standard addition employing acetone- and MeOH-PPT showed comparable recoveries for all B-vitamins but FAD, which revealed 8-9x lower concentrations with acetone, indicating only a partial recovery of milk FAD. Hence, MeOH was chosen for PPT due to overall good analyte recoveries and higher volatility and quicker evaporation than IPA.

### 3.4 Chromatography effluent diversion

Post-column lactose removal using a switch-valve was tested as an alternative. We have already described various conditions for optimal analyte resolution [2], which resulted in co-elution of lactose with some B-vitamins, such as pyridoxal, the main B6-vitamer in milk (data not shown). Here, we focused on gradient optimization for chromatographic separation of lactose and B-vitamins. Doubling the column length and changing the initial solvent ratio from 95:5 to 99:1 optimized resolution to the main B-vitamins in milk, but pyridoxamine, a B6-vitamer commonly present in milk in marginal amounts, could not be separated from lactose (Supplemental Figures 2 and 3) and was excluded.

### 3.5 Recovery rates, precision, and accuracy

Shot-to-shot variance in ^13^C-caffeine response of 100 injections revealed a CV of 12.6%. The standard addition experiments showed recoveries 92–119% for all vitamins but FAD (81.9%; Table 3). FAD is quantified without the isotope labeled analog, thus the lower recovery is not unexpected. Being the main B2-vitamer, milk-FAD is generally present in a concentration that can tolerate the lower recovery. The nicotinamide recovery was increased (107.5%) compared to previous reports (77–80%) [2], [5], likely because the now available isotope-labeled nicotinamide allowed corrections for true losses and matrix effects.

**Table 3:** Recovery rate [%], standard deviations (SD), and mean concentrations [µg/L] of multiple B-vitamins simultaneously analyzed in a pooled human milk sample and signal to noise ratios at 1 µg/L concentrations (15 µg/L for PA) and comparison to the values used to set the Adequate Intake for infants 0–6 months.

**Vitamin**	**Recovery [%] and SD**^1^		**Mean [µg/L] and SD**^2^		**Conc. [µg/L]**	S/**N**^3^**Mean and SD**	**x-fold**^4^
Thiamin	95.9	± 5.22	47.4	± 2.31	1.0	272 ± 24	210
Riboflavin	99.3	± 9.60	459	± 27.7	1.0	188 ± 9.5	350
FAD	81.9	± 8.30	364	± 39.7	1.0	10 ± 0.8	350
Nicotinamide	107.5	± 9.99	164	± 22.5	1.0	353 ± 21	1800
Pantothenic acid	92.9	± 5.86	4791	± 238	15.0	1861 ± 117	130
Pyridoxal	118.6	± 9.81	403	± 23.7	1.0	812 ± 67	130
Pyridoxine	97.9	± 11.7	1.83	± 0.17	1.0	67 ± 5.6	6
Biotin	109.8	± 10.4	12.5	± 1.28	1.0	330 ± 2.1	145

1 Average recoveries of 5 recovery experiment using 3 level recoveries on 5 different days (n = 25).

2 Average concentrations (n = 200) of pooled milk sample over 2 months of analyses. FAD: flavin adenine dinucleotide.

3 S/N: signal to noise ratio (replicates of 3).

4 x-fold higher sensitivity of the given concentration compared to values used to set Adequate Intake (AI). FAD: flavin adenine dinucleotide.

Analyzing 200 HMPs over two months showed an inter-day variability of 4.9–13.7%. All vitamins were quantifiable above their lowest standard concentration; thus all vitamins were analyzable up to 1800-fold lower concentration than those used to set the Adequate Intake for infants 0–6 months [12].

### 3.6 Matrix effects

ME, RE, and PE were unaffected by injection volume for thiamin. Biotin’s ME, PE, and RE were significantly higher with lower injection volumes (p < 0.05, Table 4), unlike for riboflavin, nicotinamide, and pyridoxal. Pantothenic acid’s ME was least affected at lower injection volumes but with increasing effects on PE. Compared to published data [2], we found significant improvements for ME and PE, but lower RE for riboflavin and pyridoxal (p < 0.05, Table 4), possibly due to the differences in sample preparation.

**Table 4:** Matrix effect (ME), process efficiency (PE), and recovery (RE) of the stable isotope labeled internal standards in human milk at different levels of injection volume (mean ± SD).

**Vitamin**	**Effect**	**3 µL**^1^	**5 µL**	**10 µL**	**Ref**[2]^2^
^13^C_4_-thiamin	ME, % ± SD	114 ± 5.3	112 ± 6.4	110 ± 5.0	36.3 ± 4.9*
	PE, % ± SD	72 ± 5.2	71 ± 4.9	74 ± 3.9	25.2 ± 1.1
	RE, % ± SD	82 ± 3.2	79 ± 3.0	81 ± 3.4	69.5 ± 3.1
^13^C_4_,^15^N_2_-riboflavin	ME, % ± SD	114^a^ ± 0.9	97^b^ ± 2.7	79^c^ ± 4.4	61.3 ± 2.9*
	PE, % ± SD	75 ± 0.8	76 ± 2.3	74 ± 3.9	53.8 ± 0.8*
	RE, % ± SD	85^a^ ± 1.1	74^b^ ± 1.2	58^c^ ± 7.3	87.9 ± 1.3*
^13^C_6_-nicotinamide	ME, % ± SD	95^a^ ± 4.7	79^b^ ± 2.0	69^c^ ± 1.5	n/a
	PE, % ± SD	73 ± 3.5	74 ± 1.1	75 ± 2.6	n/a
	RE, % ± SD	69^a^ ± 0.8	58^b^ ± 3.6	52^c^ ± 1.8	n/a
^13^C_6_,^15^N_2_-pantothenate	ME, % ± SD	100^a^ ± 9.1	82^b^ ± 5.1	82^b^ ± 12.8	n/a
	PE, % ± SD	70^b^ ± 6.0	82^a^ ± 3.4	82^a^ ± 3.2	n/a
	RE, % ± SD	69 ± 2.0	67 ± 3.2	67 ± 12.5	n/a
^2^H_3_-pyridoxal	ME, % ± SD	80^a^ ± 3.9	64^b^ ± 1.3	54^c^ ± 1.1	9.0 ± 1.1*
	PE, % ± SD	73 ± 4.6	72 ± 2.1	70 ± 2.7	6.0 ± 0.3*
	RE, % ± SD	58^a^ ± 1.1	46^b^ ± 1.8	38^c^ ± 1.6	68.8 ± 3.2*
^2^H_2_-biotin	ME, % ± SD	139^a^ ± 4.1	129^b^ ± 3.5	113^c^ ± 4.7	n/a
	PE, % ± SD	76^a^ ± 2.8	76^a^ ± 1.3	70^b^ ± 3.4	n/a
	RE, % ± SD	106^a^ ± 4.1	98^b^ ± 3.0	79^c^ ± 2.2	n/a

1 Injection volume.

2 injection volume used: 10 µL. P-values were obtained by general linear models when examining the results obtain with different injection volumes 3, 5, and 10 µL in this study. Labeled means (n = 6) in a row without common letter differ. Differences between results published in reference 2 and the 10 µL injection were tested by Student’s *t*-test, and indicated by asterisks. Statistical differences were observed when p < 0.05. SD: standard deviation.

### 3.7 Analyte stability during sample preparation

**Fig. 1:**
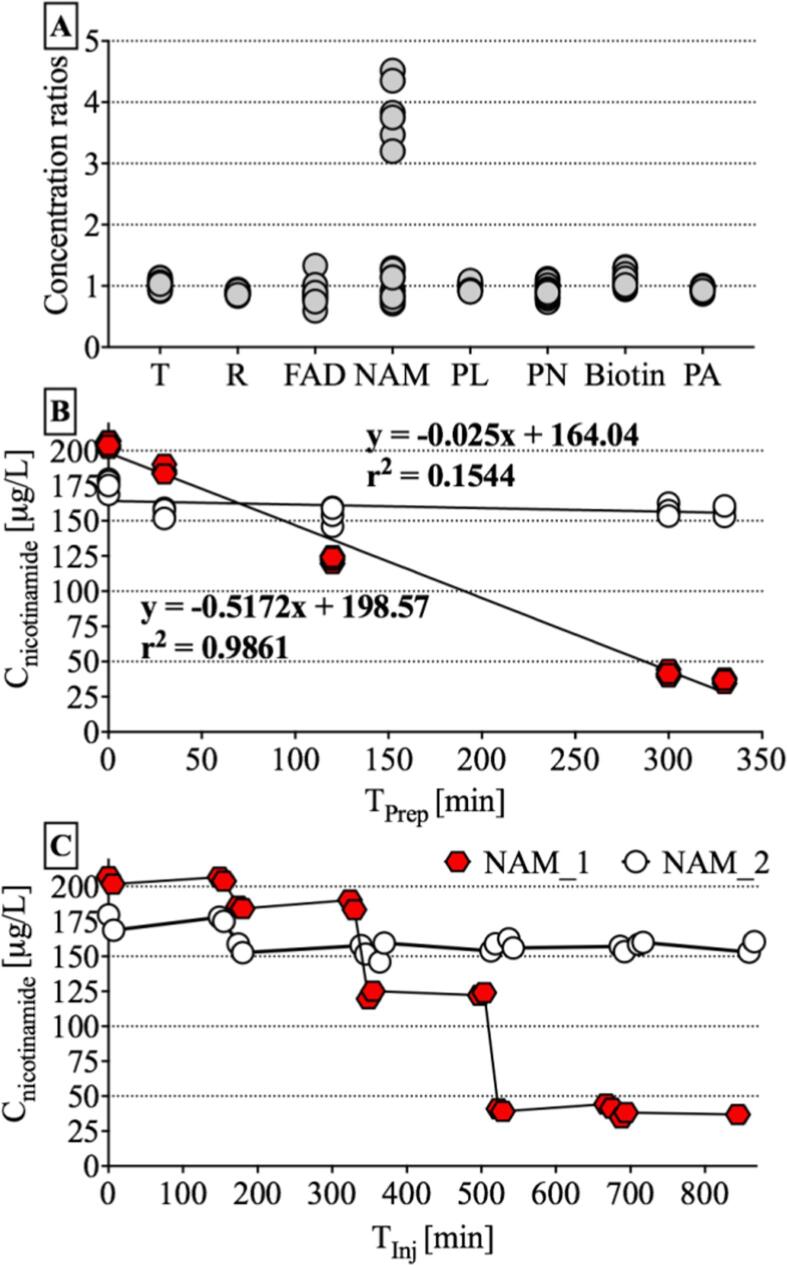
Concentration ratios of B-vitamins in pooled human milk sample aliquots. (A): frozen aliquots thawed with samples prior to sample preparation vs. aliquots sourced from HMP stored on ice throughout the day, (B): nicotinamide concentrations of HMP based on time of sample preparation, and (C) nicotinamide concentrations based on time of injection. Two HMP aliquots were prepared in a sample set of 24 (2 HMPs + 22 samples). A total of 5 sets were prepared per day. Both HMP aliquots were injected at the beginning and the end of each set (Fig. 1C) (FAD: flavin adenine dinucleotide, HMP: human milk pool, NAM: nicotinamide, NAM_1: nicotinamide concentrations obtained from HMP stored on ice during sample preparation, NAM_2: nicotinamide concentrations observed from HMP aliquots thawed immediately prior to sample preparation, PA: pantothenic acid, PL: pyridoxal, PN, pyridoxine, R: riboflavin, T: thiamin, T_Inj_: time of injection (first injection = 0 min), T_Prep_: time of sample preparation (start of first set = 0 min)).

The ratios of frozen HMPs to those stored on ice revealed a random distribution around 1.0 for all vitamins but nicotinamide (Fig. 1A). The 4–5-fold higher concentrations in the frozen samples suggested analyte degradation in thawed samples on ice. Plotting nicotinamide concentrations against the time of sample preparation (Fig. 1B) and injection (Fig. 1C) showed a linear and stepwise, respectively, decreasing relationship, which occurred with every new sample set prepared, but not between the technical HMP replicates of one set. Thus, nicotinamide degrades most likely on ice, most severely in the first 5 h as sets prepared later revealed comparable concentrations.

The sample extract was stable for at least 15 h at 10 °C; re-injection of all HMP extracts at the end of the analytical run of 5 sets on 2 different days (n = 54) revealed an intra- and inter-day variability < 9.0%. Frozen HMPs showed an inter-day variability for all analytes < 13.7%, including nicotinamide (Table 4). This degradation was specific for endogenous nicotinamide and not found for the added ^13^C_6_-nicotinamide-IS, which should undergo similar changes as nicotinamide, e.g. conversion into other B3-vitamers. Thus, the reason for this phenomenon remains unknown. Nonetheless, the linear fashion of this degradation allows for post-run concentrations adjustments, but QC samples should be thawed with the sample set to avoid mathematical adjustments.

The changes described here allowed for the analysis of at least 120 samples, a significant increase compared to our previous report [2], indicative of improved stability and robustness of the method for sample and system.

## 4 Conclusions

Previous reports on human milk B-vitamin analysis by LC-MS have not examined the impact of endogenous lactose on the equipment and analysis. This is the first report focusing on targeted removal of known matrix interferences from human milk and on the effects of remaining matrix constituents. We have described the inefficiency of commonly used sample preparation techniques as well as an efficient post-column removal of milk lactose and possibly other polar interferences. This chromatographic separation of matrix from analytes allows for a quicker sample preparation and can be routinely incorporated into the analytical run, resulting in a more robust method with lower matrix effects, which now allows for higher-throughput without loss of sensitivity.

**Author Statement**

The contribution of all authors are as shown below:

Conceptualization: Daniela Hampel (DH), John W. Newman (JWN), Setareh Shahab-Ferdows (SSF)

Formal analysis: DH

Funding acquisition: Lindsay H. Allen (LHA)

Methodology: DH, JWN

Validation: DH

Original manuscript draft: DH

Draft revision: SSF, JWN, LHA

Editing and revisions: DH.

## Declaration of Competing Interest

The authors declare that they have no known competing financial interests or personal relationships that could have appeared to influence the work reported in this paper.
