# Supplementary material for: Improving LC-MS analysis of human milk B-vitamins by lactose removal
Source: J Chromatogr B Analyt Technol Biomed Life Sci. 2021 Oct 15;1183:122968. doi: 10.1016/j.jchromb.2021.122968 (PMC8752959; doi:10.1016/j.jchromb.2021.122968)
Supplement: Supplementary data 1 [file mmc1.docx]

**Supplemental Table 1:** Chemicals and Materials^1^

| **Fisher Scientific** | **Sigma-Aldrich** | **Cambridge Isotope** | **Waters** |
| --- | --- | --- | --- |
| water (LC-MS) | thiamin hydrochloride | Caffeine-trimethyl-^13^C_3_ | HPLC screw-cap amber vials |
| acetone (LC-MS) | riboflavin |  | LC-vial caps (pre-slit, PTFE/silicone) |
| acetonitrile (LC-MS) | nicotinamide |  | glass inserts (150µL) |
| 2-propanol (LC-MS) | flavin adenine dinucleotide |  | OASIS® HLB 1cc Vac Cartridge (10mg sorbent) |
| methanol (LC-MS) | pyridoxal hydrochloride |  |  |
| methyl *tert*-butyl ether (LC-MS) | pyridoxine |  |  |
| α-D-(+)-lactose | calcium pantothenate |  |  |
| 1.5mL centrifuge tubes | biotin |  |  |
| 4mL screw thread vials (15x45mm) | thiamin-(4methyl-^13^C-thiazol-5-yl-^13^C_3_) hydrochloride (IS for thiamin) |  |  |
| Ammonium formate | nicotinamide-^13^C_6_ (IS for nicotinamide) |  |  |
| Ultrafree centrifugal filters Durapore® PVDF 0.1µm | riboflavin-dioxopyrimidine-^13^C_4_^15^N_2_ (IS for riboflavin and FAD) |  |  |
|  | pyridoxal-methyl-d_3_ hydrochloride |  |  |
|  | calcium pantothenate-(*di*-β-alanine-^13^C_6_,^15^N_2_) (IS for pantothenic acid) |  |  |
|  | biotin-(ring-6,6-d_2_) (IS for biotin) |  |  |
|  | acetic acid |  |  |

^1^Fisher Scientific (Fair Lawn, NJ, USA); Sigma-Aldrich (St. Louis, MO, USA); Cambridge Isotope Laboratories (Andover, MA, USA); Waters (Milford, MA, USA) (FAD: flavin adenine dinucleotide; IS: internal standard).

**Supplemental Table 2:** MS/MS ion transitions of 8 B-vitamins, their internal standard, and system control in positive ion mode

| **Analyte** |  |  | | **QTRAP** | | **Quattro-Micro** | |
| --- | --- | --- | --- | --- | --- | --- | --- |
|  | **RT** | **MRM** | | **DCP** | **CE** | **Cone** | **Collision** |
|  | *[min]* |  |  | *[V]* | *[eV]* | *[V]* | *[V]* |
| **Analytes** |  |  |  |  |  |  |  |
| Pantothenate | 2.03 | 220.1 | > 202.0 | 75 | 20 | n/a | n/a |
| Pyridoxal | 2.14 | 168.0 | > 150.0 | 55 | 21 | 25 | 10 |
| Pyridoxine | 2.20 | 170.2 | > 134.3 | 42 | 31 | 25 | 10 |
| Thiamin | 2.21 | 265.0 | > 122.0 | 45 | 21 | 30 | 15 |
| Nicotinamide | 2.30 | 123.0 | > 80.0 | 65 | 30 | 35 | 20 |
| Biotin | 2.32 | 245.2 | > 227.4 | 50 | 21 | n/a | n/a |
| FAD | 2.41 | 786.3 | > 348.0 | 90 | 33 | 30 | 25 |
| Riboflavin | 2.67 | 377.0 | > 243.0 | 70 | 36 | 40 | 25 |
| **Standards** |  |  |  |  |  |  |  |
| ^13^C_6_,^15^N_2_-pantothenate | 2.03 | 224.1 | > 206.0 | 75 | 20 | n/a | n/a |
| ^2^H_3_-pyridoxal | 2.14 | 153.0 | > 97.0 | 80 | 27 | 40 | 20 |
| ^13^C_4_-thiamin | 2.20 | 269.0 | > 122.0 | 45 | 21 | 25 | 10 |
| ^2^H_2_-biotin | 2.30 | 247.2 | > 229.4 | 50 | 21 | n/a | n/a |
| ^13^C_6_-nicotinamide | 2.30 | 129.1 | > 85.1 | 65 | 30 | n/a | n/a |
| ^13^C_4_^,15^N_2_-riboflavin | 2.67 | 383.0 | > 249.0 | 70 | 36 | 30 | 25 |
| ^13^C_3_-caffeine | 2.71 | 198.0 | > 140.0 | 65 | 27 | 30 | 20 |

RT: retention time, MRM: multiple reaction monitoring, DCP: declustering potential, CE: collision energy.

**Supplemental Table 3:** Global MS settings in positive ion mode

| **Parameters for UPLC-MS/MS** | | **Parameters for HPLC-MS/MS** | | **Lactose** |
| --- | --- | --- | --- | --- |
| ***QTRAP*** | | ***Micro-Quattro*** | |  |
| Collision gas | medium | Capillary voltage | 3.3kV | 3.0kV |
| Curtain gas flow | 20mL/min | Desolvation temperature | 375°C | 400°C |
| Ion spray voltage | 5500V | Desolvation gas flow | 750L/h | 650L/h |
| Turbo gas temperature | 550°C | Source temperature | 125ºC | 125ºC |
| Ion source gases 1/2 | 40psi | Cone gas flow | 25L/h | 24L/h |
| Entrance potential | 10V | Entrance voltage | -1V | 50V |
| Collision cell exit potential | 10V | Exit voltage | 2V | 50V |
| Dwell time | 100ms | Dwell time | 0.08s | 2.0s |
| Interface heater | on | Multiplier | 500V | 650V |
|  |  | Cone voltage (full scan) | n/a | 25V |
| ***UPLC*** |  | ***HPLC*** |  |  |
| AS temperature | 10ºC | AS temperature | 10ºC |  |
| Column temperature | 40ºC | Column temperature | 40ºC |  |
| Flow rate | 0.25mL/min | Flow rate | 0.35mL/min |  |
| Solvent A | 10mM NH4-F_aq_ | Solvent A | 10mM NH4-F_aq_ |  |
| Solvent B | ACN | Solvent B | ACN |  |
| Gradient:  0min  1min  2-2.5min  2.6-4min | 99% A  85% A  40% A  99% A | Gradient:  0-1min  2-2.5min  3.5min  4-5.5min | 97% A  50% A  80% A  97% A |  |

UPLC-MS/MS: ACQUITY UPLC (Waters; Milford, MA, USA) - API 4000 QTRAP MS/MS (AB Sciex; Foster City, CA, USA), HPLC-MS/MS: Alliance 2695 HPLC system - Micromass Micro Quattro mass spectrometer (Waters; Milford, MA). AS: autosampler, NH_4_-F_aq_: ammonium formate in aqueous solution.

**Supplemental Table 4:** Standard preparation scheme^1^

| **Analyte** | **Internal Standard** | **Stock [**µg/mL] |
| --- | --- | --- |
| Thiamin | ^13^C_4_ -thiamin | 72.7 |
| Riboflavin | ^13^C_4_,^15^N_2_-riboflavin | 10.5 |
| FAD | ^13^C_4_,^15^N_2_-riboflavin | 59.8 |
| Nicotinamide | ^13^C_6_-nicotinamide | 125.0 |
| Pyridoxal | ^2^H_3_-pyridoxal | 80.0 |
| Pyridoxine | ^2^H_3_-pyridoxal | 80.0 |
| Biotin | ^2^H_2_-biotin | 220.0 |
| Ca-pantothenate | ^5^N_2_-Ca-pantothenate | 120.0 |
| ^13^C_4_ -thiamin | ----- | 110.0 |
| ^13^C_4_,^15^N_2_-riboflavin | ----- | 100.0 |
| ^13^C_6_-nicotinamide | ----- | 100.0 |
| ^5^N_2_-Ca-pantothenate | ----- | 160.0 |
| ^2^H_3_-pyridoxal | ----- | 100.0 |
| ^2^H_2_-biotin | ----- | 240.0 |
| ^13^C_3_-caffeine | ----- | 100.0 |

^1^Riboflavin, FAD, nicotinamide, calcium pantothenate, and biotin stock solutions were stored at -70°C while the remaining stock solutions were kept at 0°C (Ca: calcium; FAD: Flavin adenine dinucleotide).
