# Supplementary material for: Improving LC-MS analysis of human milk B-vitamins by lactose removal
Source: J Chromatogr B Analyt Technol Biomed Life Sci. 2021 Oct 15;1183:122968. doi: 10.1016/j.jchromb.2021.122968 (PMC8752959; doi:10.1016/j.jchromb.2021.122968)

**Supplemental Figure 1:** Macronutrient contribution ratios (water, protein, fat, sugar) of human urine, plasma, and milk.


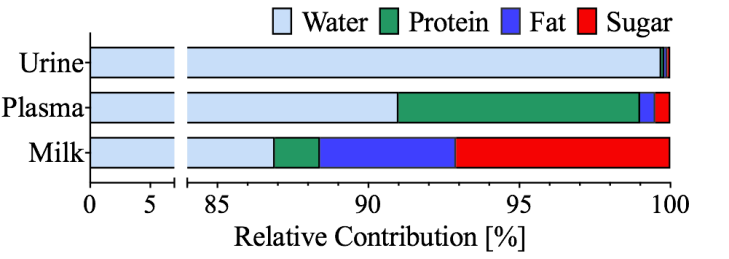


**Supplemental Figure 2:** Combined extracted ion chromatograms (XIC) of lactose (chemical structure provided), pyridoxamine (co-eluting with lactose), and B-vitamins analyzed using the method described. Dashed line indicated the diverter valve switching from waste to MS at 1.8min.


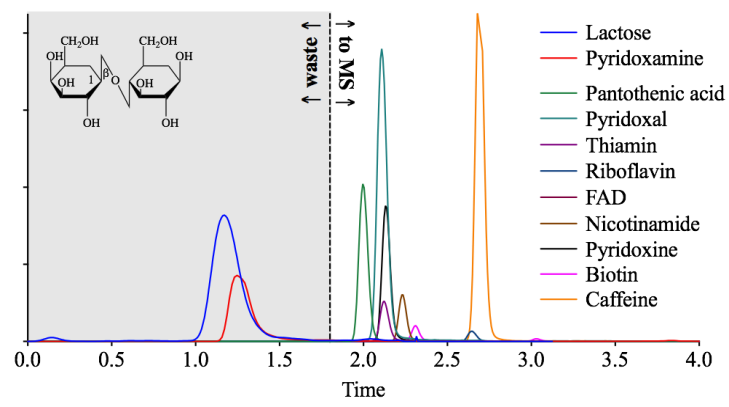


Lactose: XIC of Q1: 341.2-341.3 (M^-^). MRM for B-vitamins are described in Supplemental Table 1.

**Supplemental Figure 3:** Extracted ion chromatograms of (a) thiamin, (b) riboflavin, (c) flavin adenine dinucleotide (FAD), (d) nicotinamide, (e) pyridoxal, (f) pyridoxine, (g) biotin, (h) calcium pantothenate, and (i) ^13^C_3_-caffeine in a standard mix (50µg/L).


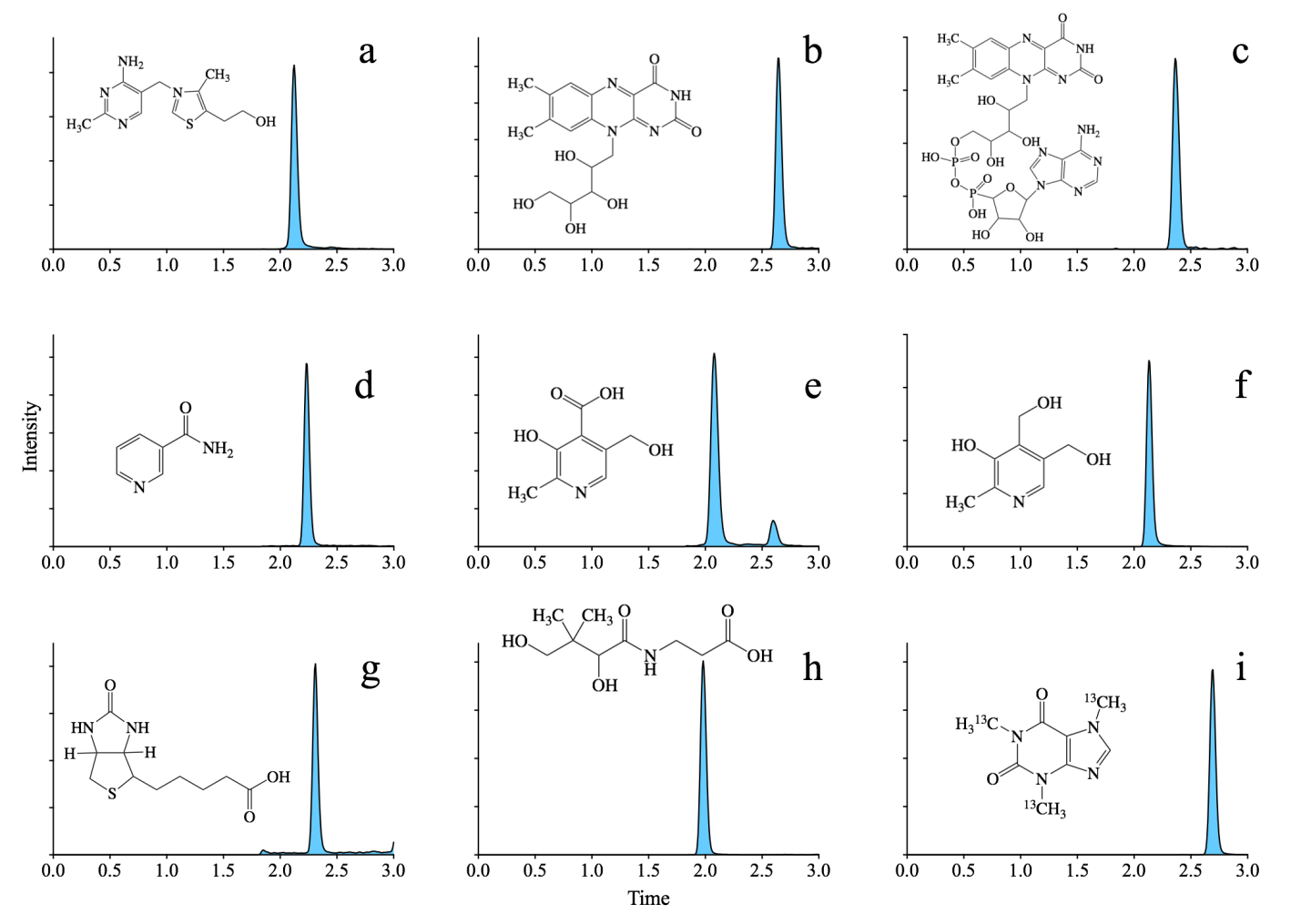

Supplement: Supplementary data 2 [file mmc2.docx]
